# Supplementary material for: Therapeutic Switching of Rafoxanide: a New Approach To Fighting Drug-Resistant Bacteria and Fungi
Source: Microbiol Spectr. 2023 Jul 17;11(4):e02679-22. doi: 10.1128/spectrum.02679-22 (PMC10433953; doi:10.1128/spectrum.02679-22)
Supplement: Supplemental file 5 — Supplemental material. Download spectrum.02679-22-s0005.pdf, PDF file, 0.8 MB [file spectrum.02679-22-s0005.pdf]

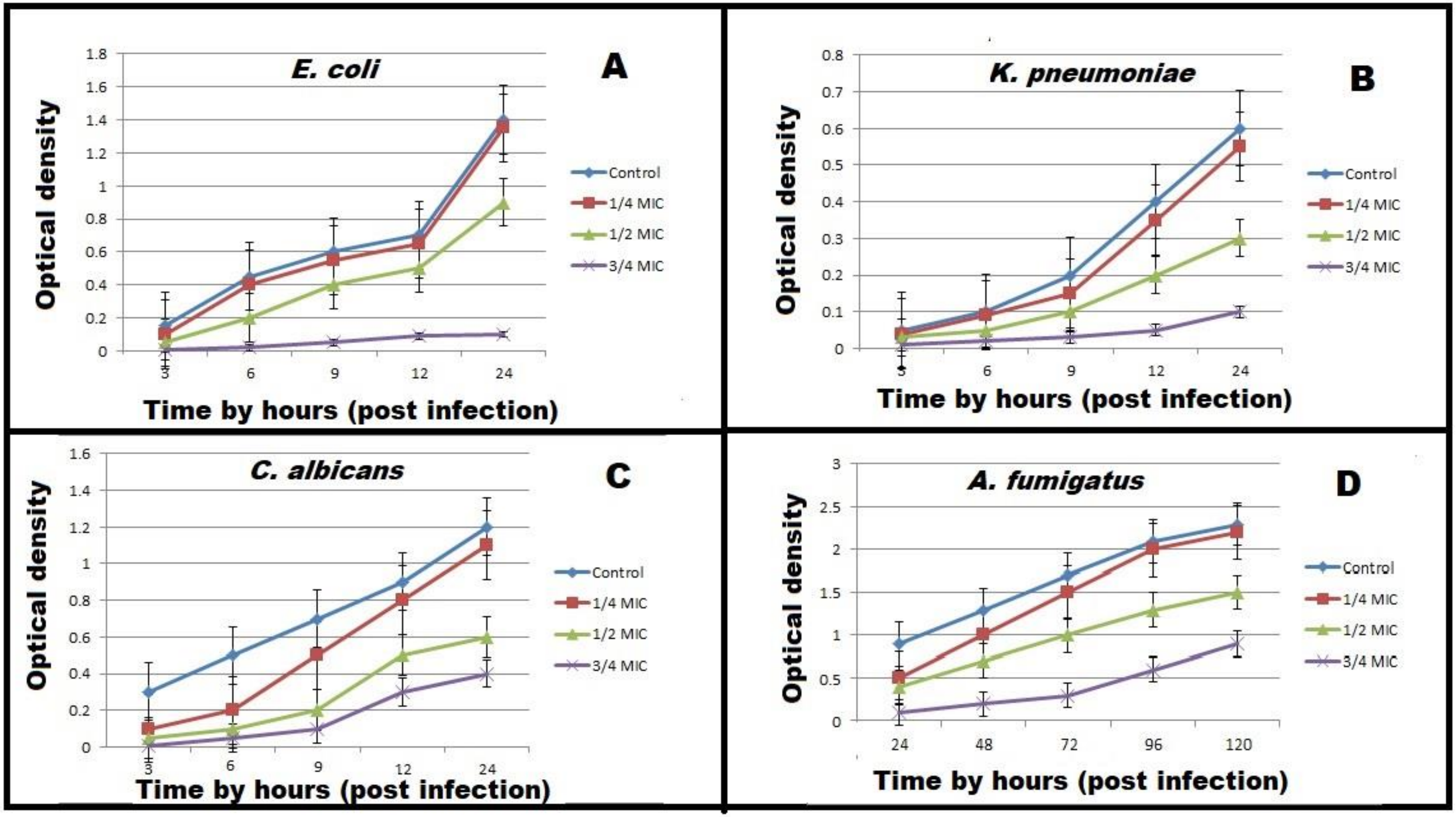

Supplementary Fig.1: Growth curve analysis of representative *Escherichia coli*, *E. coli* (A), *Klebsiella pneumonia*, *K. pneumoniae* (B), *Candida albicans*, *C. albicans* (C) and *Aspergillus fumigatus*, *A. fumigatus* (D) isolates cultured in Muller-Hinton broth in the absence (control) or presence of 1/4, 1/2 and 3/4× MIC rafoxanide concentrations obtained by plotting optical density at 600 nm, OD_{600} values versus time (hours) post infection with correspondent standard error. Mean values are representative of three independent biological replicates.

MIC: minimum inhibitory concentration

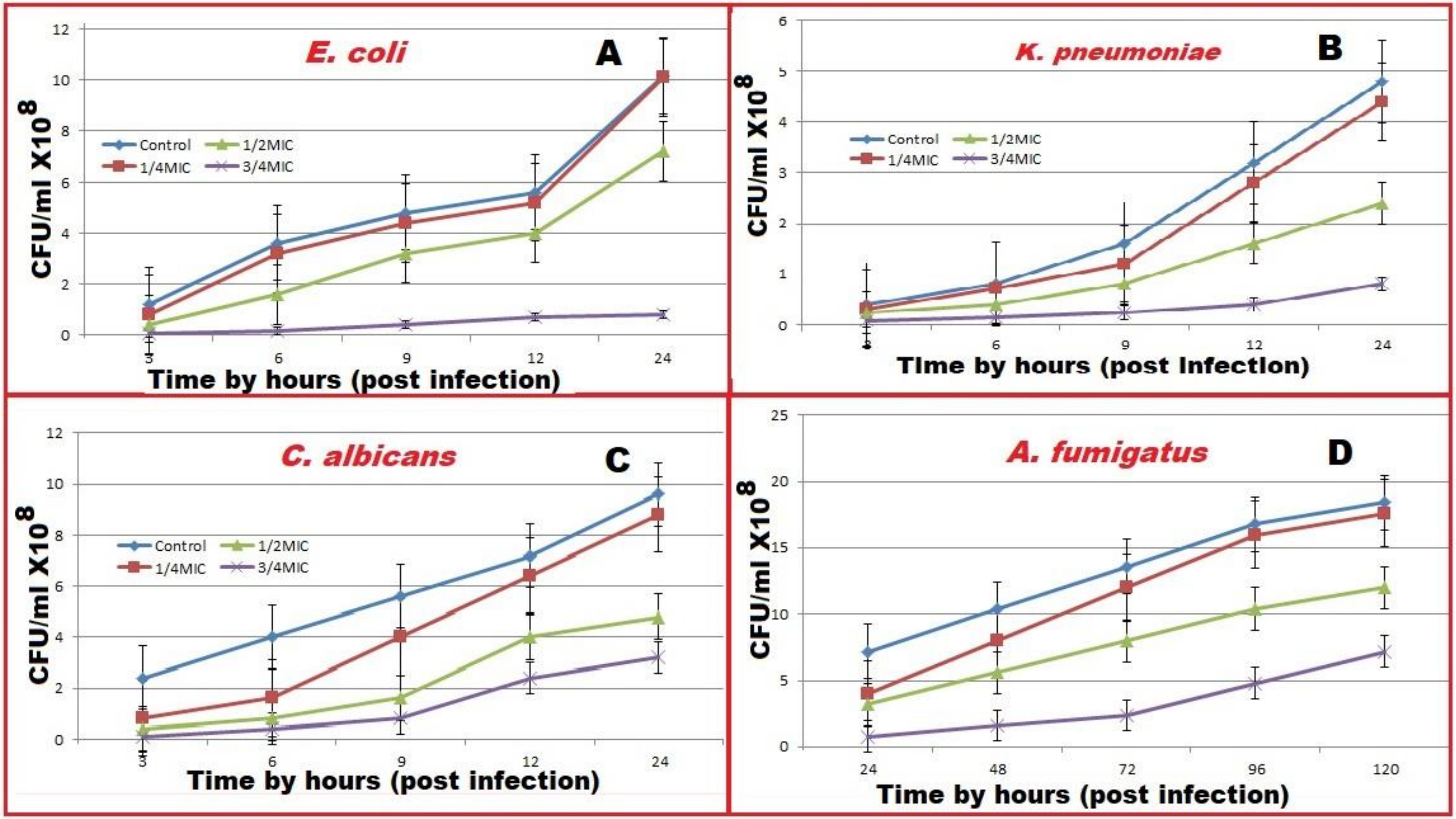

Supplementary Fig.2: Growth curve analysis of representative *Escherichia coli*, *E. coli* (A), *Klebsiella pneumonia*, *K. pneumoniae* (B), *Candida albicans*, *C. albicans* (C) and *Aspergillus fumigatus*, *A. fumigatus* (D) isolates cultured in Muller-Hinton broth in the absence (control) or presence of 1/4, 1/2 and 3/4× MIC rafoxanide concentrations obtained by plotting CFU/mL values versus time (hours) post infection. Mean values are representative of three independent biological replicates.

MIC: minimum inhibitory concentration, CFU: colony forming unit

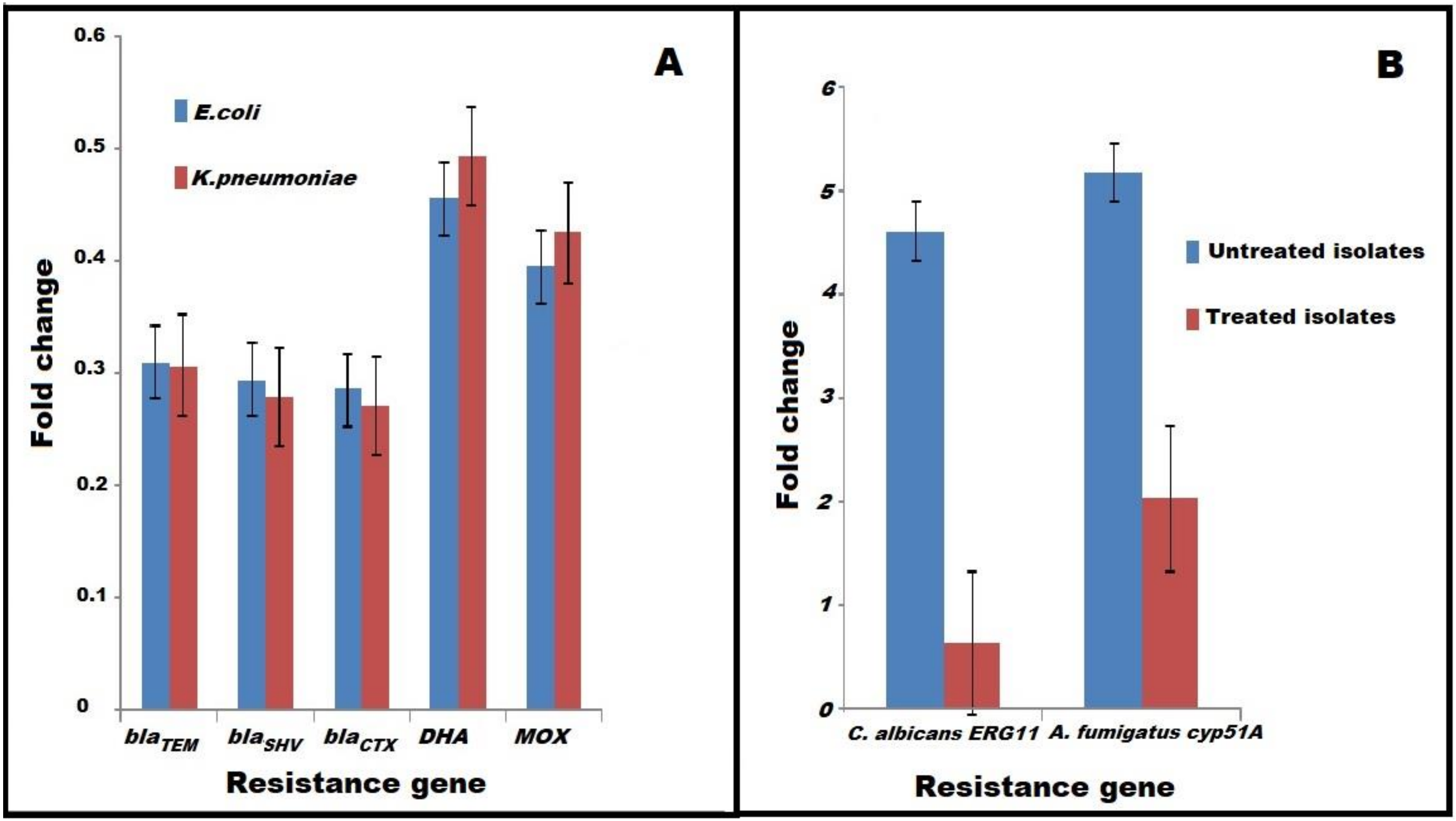

Supplementary Fig.3: The mean fold changes of *bla*$_{TEM\ 1}$, *bla*$_{SHV}$, *bla*$_{CTX\text{-}M\text{-}1}$, *DHA* and *MOX* resistance genes of *Escherichia coli* (*E. coli*) and *Klebsiella pneumoniae* (*K. pneumonia*) isolates treated with rafoxanide (A) and those of *Candida albicans* (*C. albicans*) *ERG11* and *Aspergillus fumigatus* (*A. fumigatus*) *cyp51A* resistance genes of the untreated and rafoxanide-treated fungal isolates (B).
